# Biomarkers of Traumatic Brain Injury: Temporal Changes in Body Fluids

**DOI:** 10.1523/ENEURO.0294-16.2016

**Published:** 2016-12-21

**Authors:** Harel Adrian, Kvist Mårten, Nuutinen Salla, Välimaa Lasse

**Affiliations:** Medicortex Finland Oy, Itäinen Pitkäkatu 4 B, 20520 Turku, Finland

**Keywords:** biomarker, diagnostics, TBI, traumatic brain injury

## Abstract

Traumatic brain injuries (TBIs) are caused by a hit to the head or a sudden acceleration/deceleration movement of the head. Mild TBIs (mTBIs) and concussions are difficult to diagnose. Imaging techniques often fail to find alterations in the brain, and computed tomography exposes the patient to radiation. Brain-specific biomolecules that are released upon cellular damage serve as another means of diagnosing TBI and assessing the severity of injury. These biomarkers can be detected from samples of body fluids using laboratory tests. Dozens of TBI biomarkers have been studied, and research related to them is increasing. We reviewed the recent literature and selected 12 biomarkers relevant to rapid and accurate diagnostics of TBI for further evaluation. The objective was especially to get a view of the temporal profiles of the biomarkers’ rise and decline after a TBI event. Most biomarkers are rapidly elevated after injury, and they serve as diagnostics tools for some days. Some biomarkers are elevated for months after injury, although the literature on long-term biomarkers is scarce. Clinical utilization of TBI biomarkers is still at a very early phase despite years of active research.

## Significance Statement

Traumatic brain injury (TBI) is a common problem, called a “silent epidemic” because of a general unawareness of the condition. TBI is difficult to diagnose with imaging techniques, and there is no definite laboratory test to support the diagnosis. An undiagnosed case of TBI can result in premature “return to play” with severe consequences or in a chronic neurodegenerative condition later in life. An ideal laboratory test, detecting a brain injury–specific biomarker in one of the body fluids, would confirm or rule out the TBI, predict the outcome, and indicate when recovery is complete. This article reviews recent research on brain injury biomarkers that could be used for rapid and accurate diagnostics of TBI in easily accessible fluid samples.

## Introduction

Traumatic brain injury (TBI) is caused by a blow to the head, penetration of foreign objects through the skull, or sudden motions of the head. A recent systematic review reports that the overall incidence rate of TBI is 262 in 100,000, the mortality rate is 10.5 in 100,000 in Europe, and falls and road traffic accidents are the most common causes of TBI (Peeters et al., 2015). The statistics of the Centers for Disease Control and Prevention show that the overall incidence rate of TBI in the United States is 577 in 100,000 (total 1.7 million cases per year), and the mortality rate is 17.6 in 100,000 (>51,000 deaths per year; Faul et al., 2010). It is estimated, however, that the presented numbers probably underestimate the incidence of mild TBI (mTBI), and the data are confounded by the great variation in the definitions of TBI.

The pathophysiology of TBI varies considerably depending on the location of the injury, the type of injury, and its severity. A mild injury may just cause a feeling of discomfort, headache, dizziness, or transient unconsciousness, whereas moderate or severe injuries may lead to diffuse axonal injury, epidural or subdural hematomas, intracerebral bleedings, large destruction of the brain tissue, and even death (Pearn et al., 2016).

Currently the diagnosis of TBI is made mainly based on a neurological examination of the patient and additionally using imaging radiology techniques such as computed tomography (CT) or magnetic resonance imaging (MRI). The Glasgow Coma Scale (GCS) assesses the severity of TBI on the basis of cognitive behavior (Teasdale and Jennet, 1974; Teasdale et al., 2014). A total score of 13–15 refers to mTBI, 9–12 to moderate TBI, and 3–8 to severe TBI (Faul and Coronado, 2015). Imaging techniques do not provide definitive means for the diagnostics of TBI, since they fail to find alterations in a large proportion of patients that have a mild to moderate injury (Hofman et al., 2001; Borg et al., 2004; Hughes et al., 2004; Belanger et al., 2007). One of the more advanced modes of MRI currently is diffusion tensor imaging (DT-MRI). It traces the direction of water molecules’ diffusion and uses computed parameters of diffusivity as measures of axonal integrity (Delouche et al., 2016). The technique allows for accurate 3D modeling of neural tracts (tractography) by means of computerized image analysis. DT-MRI is considered a promising tool for TBI diagnostics because of the ability to focus on axonal structures, but the literature regarding the detection of acute mTBI is somewhat inconsistent. For example, Arfanakis et al. (2002) and Inglese et al. (2005) reported significant alterations in diffusivity after mTBI in particular brain areas, implying diffuse axonal injury (DAI), whereas Ilvesmäki et al. (2014) concluded that acute mTBI is not associated with white matter change on DT-MRI. Another special modality of MRI is functional MRI (fMRI), which indicates the activation of various brain regions upon different stimuli or tasks. The imaging detects changes in cerebral blood flow and oxygen consumption based on different magnetic properties between oxyhemoglobin and deoxyhemoglobin. In the diagnostics of mTBI, fMRI may be a promising technology. It has shown functional alterations in the brain of concussed athletes who were asymptomatic in clinical assessment and neuropsychological testing (Slobounov et al., 2011), and subtle changes have been detected even 1 year after an injury (McAllister et al., 2006). However, the literature regarding the diagnostics of acute mTBI using fMRI is scarce (McDonald et al., 2012).

**Figure 1.**
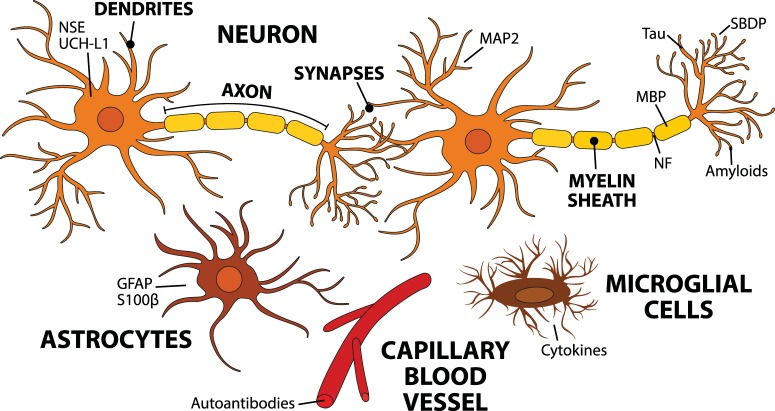
Biomarkers detected after TBI. This schematic figure demonstrates the possible cellular origin of the biomarkers that are associated with TBI pathology. TBI causes cellular injury to neuronal and nonneuronal cells. The trauma manifests in damaged BBB, ionic imbalances, energy depletion, and cell death. The cascade of events starts by an increase in extracellular glutamate and intra-axonal calcium levels. Increased calcium activates calpains, caspases, and phosphatases that trigger the cleavage of NFs and α-spectrin, which leads to the disruption of the cytoskeleton and cell death. Calcium also activates transcription factors that upregulate inflammatory mediators, such as TNF-α and IL-1β. In addition, mechanical injury causes synaptic dysfunction and accumulation and release of intracellular products, which impairs neurotransmission.

Biomarkers of a brain injury (Fig. 1) can be detected in the cerebrospinal fluid (CSF) and in the blood directly after TBI (Zetterberg and Blennow, 2015). The blood–brain barrier (BBB), which normally is almost impermeable, can lose its integrity upon brain injury and allow the permeation of molecules into the blood (Başkaya et al., 1997). Alternatively, they may be transported to the blood via the glymphatic system (Plog et al., 2015). Urine is sampled noninvasively and can be an appropriate sample source in decentralized field assay conditions. The route of biomarkers from the brain to urine is indirect and contains potential barriers and dilutive interfaces, yet markers of brain injury have been found in urine (Rodríguez-Rodríguez et al., 2012; Ottens et al., 2014; Oliver et al., 2015).

Recent review articles discuss the biomarkers of TBI from various viewpoints, for example, comparison of body fluids as a source of biomarkers, their diagnostic and prognostic value, and the use of biomarkers in special situations such as sports and military accidents (Jeter et al., 2013; Yokobori et al., 2013; Zetterberg et al., 2013; Strathmann et al., 2014; Kulbe and Geddes, 2016). The timeline, or kinetics, of the emergence, persistence, and decline of the biomarkers is a rising area of active research. Acute biomarkers are valuable for the confirming or ruling out a brain injury shortly after a head injury. On the other hand, persistent biomarker levels can reveal a past TBI event. This information can help a person to avoid risky behavior that may result in a new head injury. It can also provide evidence for juridical processes and insurance claims related to an accident in which a head injury has occurred. In this review, we briefly introduce and discuss recent research and temporal courses studied on TBI biomarkers, focusing on body fluid samples that are easily accessible for rapid and specific diagnostics.

## Biomarkers

### Biomarkers of TBI in body fluids

#### S100β

S100β is one member of the calcium binding protein family S100, which was first isolated from the bovine brain in 1965 (Moore, 1965). A relationship between neurological injury and S100β was discovered by Michetti et al. (1980). S100β is expressed in astrocytes and other neural cells, but also in some cells of nonneural origin (summarized by Donato et al. 2009). High S100β levels correlate with mortality and unfavorable prognosis (Mercier et al., 2013). However, S100β is not brain injury specific: its concentration increases in some other diseases and traumas (Anderson et al., 2001; Undén et al., 2005; Studer et al., 2015), as well as during intensive physical exercise (Stocchero et al., 2014). A later sampling (12–36 h after trauma) of S100β has shown enhanced prognostic value over early sampling (Thelin et al., 2013). Despite compromises in brain specificity, S100β has a good negative predictive value, and it is getting attention as a clinical marker to rule out a brain injury (Undén et al., 2013).

#### S100β kinetics

A study by Rodríguez-Rodríguez et al. (2012) showed a peak in serum <6 h after injury and thereafter a gradual decrease until the end of the follow-up period (96 h). Thelin et al. (2014) reported that a secondary peak (a new rise even as low as 0.05 µg/l) detected in serum ≥48 h after trauma strongly correlated with later pathological findings in CT and MRI. A comprehensive kinetic modeling by Ercole et al. (2016) confirms that a relatively sharp peak of S100β occurs in serum just 1 day after trauma (mean time to peak, 27.2 h). S100β has also been studied in urine. A study showed a peak at admission (≤6 h postinjury) and a subsequent decrease until 48 h, after which the concentration slightly increased until 96 h (Rodríguez-Rodríguez et al., 2012). Another study in urine (pediatric patients) showed that S100β peaked at a mean of 55.3 h after injury (Berger and Kochanek, 2006). The peak in serum appeared significantly earlier, at a mean of 14.6 h after injury. Overall, the concentration of S100β in the blood rises and peaks in some hours, but then it decreases quite rapidly, since the half-life of S100β in serum is only on the order of 1.5 h (Townend et al., 2006).

#### Glial fibrillary acidic protein

Glial fibrillary acidic protein (GFAP) is an intermediate filament protein that was reported for the first time in 1971 (Eng et al., 1971), and its relation to brain injuries was elucidated later in animal studies (Latov et al., 1979; Moore et al., 1987). GFAP is abundantly expressed in the cytoskeleton of astrocytes, although some expression in other types of cells has been discovered (Kasantikul and Shuangshoti, 1989). However, several studies confirm the high specificity of GFAP to brain injuries in comparison to other biomarkers such as S100β and neuron-specific enolase (Honda et al., 2010; Papa et al., 2014, 2016b). The concentration of GFAP in serum differs between patients that have a GCS value of 3–5 and 13–15, and thus, GFAP has diagnostic potential to discriminate between severe and mild cases of TBI (Lee et al., 2015). Acute GFAP levels correlate with the recovery and outcome of the patient (Mannix et al., 2014; Takala et al., 2016), although in mTBI cases, the predictive value was found to be weaker (Metting et al., 2012).

#### GFAP kinetics

One of the earliest studies (Missler et al., 1999) measuring GFAP in human blood reported that admission samples (3–16 h postinjury) showed increased levels of blood GFAP in 12 of 25 patients, with a mean concentration of 0.10 µg/l. Approximately 85% of the healthy controls were below the detection limit of 0.010 µg/l. In 24- and 48-h samples, GFAP was detectable in a smaller number of patients, and the levels were only slightly elevated. A more recent study (Lei et al., 2015), which followed the levels of GFAP for 0–5 days after the injury, reported that the peak was detected at admission (0.5–4 h). Žurek and Fedora (2012) monitored children that had TBI, and they also found the highest levels of GFAP in the admission samples drawn <12 h after injury. The GFAP levels were much higher in nonsurvivors compared with survivors; however, the temporal profiles were similar in both groups during the 6-day follow-up period. Papa et al. (2016a) monitored GFAP levels at short intervals in patients enrolled no more than 4 h after injury. They found that GFAP was detectable in serum within 1 h, and the peak appeared at 20 h in patients who had a mild or moderate TBI. Other studies have also confirmed that GFAP is detectable in serum as early as 1 h after the injury (Papa et al., 2014, 2015b).

#### Neuron-specific enolase

Enolases are enzymes that catalyze the conversion of 2-phosphoglycerate into phosphoenolpyruvate in the glycolysis pathway. Evidence on the existence of a brain-specific enolase came forth in the 1970s (Bock and Dissing, 1975; Rider and Taylor, 1975). Known as neuron-specific enolase (NSE), γ-enolase, or enolase 2, the neuron-specific isoenzyme consists of two γ-subunits (γγ) with a total molecular weight of 78 kDa. Increased levels of NSE in the serum of TBI patients were first observed in the early 1990s (Skogseid et al., 1992). A recent meta-analysis reports that high concentrations of NSE in serum is significantly associated with mortality and unfavorable outcome (Cheng et al., 2014). A risk related to the use of NSE is that the samples may be contaminated by enolases from hemolyzed red blood cells (Ramont et al., 2005), although improved accuracy can be obtained with a correction factor (Tolan et al., 2013). The presence and diagnostic value of NSE is not clear in mTBI and concussion, however, as a significant elevation of NSE in the serum was detected after kicks to the head in karate (Graham et al., 2015) but not in concussed ice hockey players (Shahim et al., 2014).

#### NSE kinetics

Herrmann et al. (2000) reported that the temporal profiles of NSE in serum differed significantly between groups with mTBI and moderate to severe TBI, but the concentration came down to the normal level in 25–48 h even in the severe TBI group. Further, in cases of DAI and intracranial pressure, the peak of NSE appeared on the third day. Žurek and Fedora (2012) also found different severity-dependent profiles in children; whereas the concentration of NSE gradually decreased after injury in survivors, nonsurvivors had increasing NSE concentrations during days 1 and 2. A recent study analyzed serum NSE levels for 5 d after severe TBI (Olivecrona et al., 2015). The initial NSE level (sampled on average 15 h postinjury) reached ∼19 µg/l and gradually decreased to ∼8 µg/l until day 5. The study also showed an association of NSE levels with intracranial pressure, cerebral perfusion pressure, and CT findings.

#### Ubiquitin C-terminal hydrolase-L1

Ubiquitin carboxy-terminal hydrolase L1 (UCH-L1), also known as protein gene product 9.5 (PGP 9.5), is a 27-kDa enzyme abundant in the soma of neurons. UCH-L1 cleaves ubiquitin, a small regulatory protein involved in labeling proteins for metabolism, from the C terminus of its target proteins. UCH-L1 was discovered in the 1980s and constitutes some 1–5% of the brain’s total protein content (Doran et al., 1983; Wilkinson et al., 1989).

Active research on UCH-L1 in the context of TBI has emerged since the first decade of the 2000s (Siman et al., 2009; Papa et al., 2010). UCH-L1 has been shown to be a brain-specific biomarker, and its levels correlate with the severity of TBI and outcome (Mondello et al., 2012b; Takala et al., 2016). In mTBIs, the results are inconsistent; Papa et al. (2012) reported that serum UCH-L1 levels discriminate mTBIs from controls, whereas some studies were unable to show a sufficient discriminating power between patients with mTBI and noninjured controls (Berger et al., 2012; Puvenna et al., 2014). However, UCH-L1 was shown to outperform GFAP and S100β when the goal was to reduce CT scans in patients with mild to moderate TBI (Welch et al., 2016).

#### UCH-L1 kinetics

The concentration of UCH-L1 in serum rises within a few hours after injury, but the level also declines quite fast (Brophy et al., 2011; Mondello et al., 2012b). In cases of mild to moderate TBI, the concentration of UCH-L1 was shown to peak in 8 h after injury, which was earlier than the peak of GFAP (Papa et al., 2016a). The time window for the detection of UCH-L1 was short, but the authors discussed that the rapid rise of UCH-L1 enables the assaying of TBI in point-of-care settings at the accident site or in ambulances.

#### Neurofilaments

The neuronal cytoskeleton is mainly composed of neurofilaments (NFs), which is one subcategory (Type IV) of intermediate filaments. The three main proteins (NF subunits) that compose neurofilaments are named according to their sizes: light (NF-L, 68–70 kDa), medium (NF-M, 145–160 kDa), and heavy (NF-H, 200–220 kDa). Neurofilaments are localized in the axon, and they regulate the structure and diameter of a neuron (Trojanowski et al., 1986). The phosphorylated form of the heavy subunit (p-NF-H) is specific to axons and can be detected in the blood with an immunoassay, thus being a potential biomarker of DAI (Shaw et al., 2005; Anderson et al., 2008). Gatson et al. (2014) reported that the level of p-NF-H was significantly increased in the serum of mTBI patients and clearly distinguished patients from noninjured controls. It was also shown that p-NF-H is a decent predictive marker of outcome in adult TBI patients (Shibahashi et al., 2016).

#### NF kinetics

The kinetic profile of p-NF-H in serum differs somewhat from that of many other biomarkers. Although several biomarkers peak and then decline within a couple of days after injury, the concentration of p-NF-H still increases. The continuous increase was shown with a pediatric population during 6 consecutive days (Žurek and Fedora, 2012), and in another study within 4 up to 10 days after injury (Vajtr et al., 2012).

#### Myelin basic protein

Oligodendrocytes and Schwann cells produce the myelin sheath of the axons. The myelin sheath contains lipids and proteins, and the main protein component of the myelin sheath is myelin basic protein (MBP), which comprises ∼30% of myelin’s protein content. Myelination is an age-dependent process, and thus the amount of myelin in the CNS varies between children and adults (Steinman, 1996; Paus et al., 2001). The relation of MBP to TBI was discovered in the late 1970s (Thomas et al., 1978). MBP has been found to correlate specifically with clinical outcome (Yamazaki et al., 1995; Berger et al., 2005).

#### MBP kinetics

MBP can be detected already 1.5–8.0 h after injury (Yamazaki et al., 1995), but MBP peaks somewhat slower than S100β and NSE (Berger et al., 2005, 2006). Serum MBP remains elevated for even up to 2 weeks (Thomas et al., 1978). The time course of MBP was shown to be different in various types of TBI; in pediatric patients, serum MBP peaks later in inflicted TBI compared with noninflicted TBI (Berger et al., 2005, 2006). Specific temporal patterns thus may help in distinguishing brain injury induced by child abuse from accident-based brain injuries.

#### Spectrin breakdown products

Spectrin is a cytoskeletal protein that maintains cell membrane integrity and cytoskeleton structure (De Matteis and Morrow, 2000). Upon cellular injury, calpains and caspases cleave spectrin to spectrin breakdown products (SBDPs). Different SBDPs are present depending on the type of cell death and the enzymes involved in the process (Wang et al., 1998; Büki et al., 2000). A relevant SBDP for brain injuries is calpain-derived N-terminal αII-spectrin fragment (SNTF), which can be readily detected in concussions, but also in a subset of orthopedic injuries (Siman et al., 2013, 2015).

#### SBDP kinetics

In concussed ice hockey players, the concentration of serum SNTF increased above the prior measured preseason level 1 h after head injury. In persistent concussion (≥6 days), serum SNTF was increased as much as 2.5-fold above the baseline and stayed elevated from 1 h to 6 days. The average of the 12- to 36-h postinjury serum level showed the greatest accuracy in discriminating persistent concussions from milder concussions whose symptoms were alleviated within a few days (Siman et al., 2015).

#### Tau

Tau is one of the microtubule-associated proteins (MAPs) that were discovered in the 1970s (Weingarten et al., 1975; Witman et al., 1976). Tau is a 48- to 68-kDa protein that stabilizes microtubular assembly and is enriched in the axons of neurons, although it is not completely specific for the CNS (Goedert et al., 1989; Morris et al., 2011). Upon cellular injury and activation of proteases, tau is cleaved into fragments of 10–18 kDa and 30–50 kDa (cleaved tau or c-tau; Zemlan et al., 1999; Gabbita et al., 2005). In addition, injuries lead to the phosphorylation of tau, which in extreme cases results in the aggregation of hyperphosphorylated fragments (tau tangles) that are characteristic for neurodegenerative diseases such as Alzheimer’s disease and chronic traumatic encephalopathy (Šimić et al., 2016).

Clearly elevated levels of serum tau with reliable prognostic value have been reported after severe TBI (Liliang et al., 2010). In mTBI, serum tau levels were also increased, but the difference from the noninjured controls was not statistically significant (Bulut et al., 2006), and weaker prognostic values have been reported (Bazarian et al., 2006; Ma et al., 2008). However, new sensitive assay techniques have shown enhanced diagnostic performance for tau between injured and noninjured samples and an advantage for the use of tau in cases where many other biomarkers have failed to detect brain injury (Neselius et al., 2013; Shahim et al., 2014; Olivera et al., 2015; Rubenstein et al., 2015).

#### Tau kinetics

Ultrasensitive immunoassays have revealed temporal profiles of tau in blood. Among concussed ice hockey players, the highest total tau levels in plasma were measured during the first hour after a concussion, and the level declined already during the first 12 h. In addition, a trend to a second peak at 36 h after concussion was observed (Shahim et al., 2014). Phosphorylated tau remains elevated in serum longer than total tau (Rubenstein et al., 2015). Elevated levels of total tau in plasma were measured among soldiers who had suffered TBI during their deployment within the past 18 months, thus indicating that tau may serve as a long-term biomarker of an earlier TBI event (Olivera et al., 2015).

#### Microtubule-associated protein 2

Microtubule-associated protein 2 (MAP2), like tau, belongs to the family of microtubule stabilizing proteins. MAP2 is abundant in nerve cells and is believed to be specific for neurons’ dendritic injuries (Garner et al., 1988). Elevated levels of MAP2 were detected in the serum of severe TBI patients at 6 months after injury (Mondello et al., 2012a). Survivors of TBI had higher levels of MAP2 than patients that had gone into a vegetative state. The authors concluded that a severe TBI results in a chronic release of MAP2, but it is also a marker of remodeling and indicates emergence into the higher level of consciousness for TBI patients.

#### MAP2 kinetics

MAP2 is a novel biomarker of TBI, and the above 6-month time point is the only temporal data on the presence of MAP2 in human blood; it suggests that MAP2 can indicate a past TBI event. In human CSF, MAP2 was found to be elevated within 6 h after injury, and the concentration remained quite stable for at least 24 h (Papa et al., 2015a).

#### Amyloid β

Amyloid precursor protein is a cell surface receptor and a transmembrane precursor protein that is cleaved to various peptides, including amyloid β (Aβ), which is a 36- to 43-aa-long peptide abundant in amyloid plaques, characteristic of Alzheimer’s disease (Vivekanandan et al., 2011; Tharp and Sarkar, 2013). Abnormal concentrations or altered structure of Aβ is neurotoxic. Aβ plaques have been found in ∼30% of TBI patients, and TBI is considered an independent risk factor for Alzheimer’s disease (Roberts et al., 1994; Tsitsopoulos and Marklund, 2013). Immunohistochemical staining has shown that the accumulation of amyloid precursor protein in injured axons and thus Aβ could be a biomarker of diffuse axonal injury (Johnson et al., 2016).

#### Amyloid β kinetics

Using an ultrasensitive digital ELISA, Mondello et al. (2014) found that Aβ_42_ rises in the plasma within the first day after injury, and the level remains quite steady for at least 6 d after injury. In contrast, one study reported no change in the plasma Aβ_42_ level during a follow-up of up to 11 days after severe TBI (Olsson et al., 2004).

#### Cytokines

Neuroinflammation is an essential part of the secondary injury cascade after TBI. Several proinflammatory cytokines and chemokines are upregulated, and they recruit immune cells into the CNS and promote astrogliosis (Hellewell et al., 2016). The CNS inflammatory response initiates already a few minutes after injury, and proinflammatory mediators are highly elevated *in situ*, whereas anti-inflammatory cytokines remain unchanged (Frugier et al., 2010). Tuttolomondo et al. (2014) reported that tumor necrosis factor (TNF)-α, especially, plays an essential role in mediating an immune response in TBI and ischemic stroke. Interleukin (IL)-6 is considered another central mediator in neuroinflammation; increased levels of IL-6 in serum have been found after acute cerebral ischemia and correlated with poor functional and neurological outcome (Fassbender et al., 1994). Also, elevated levels of a small chemokine in plasma, chemokine CC ligand-2 (formerly monocyte chemoattractant protein 1) correlated with the severity of TBI (Ho et al., 2012).

#### Cytokine kinetics

High levels of cytokines have been measured predominantly in the CSF, where they peak within the first days after injury and where the concentrations of several cytokines are typically higher than in the blood (Kossmann et al., 1997; Csuka et al., 1999; Maier et al., 2001). However, Santarsieri et al. (2015) found several inflammation markers in significantly higher concentrations in the serum than in the CSF. Similar kinetic trends as in the CSF have been detected in the serum, i.e., peaking within the first days, and also a mild secondary rise of IL-10 in the second week (Csuka et al., 1999; Hayakata et al., 2004). Elevated levels of several cytokines in serum were measured for >3 months after a TBI, which indicate the presence of chronic post-TBI inflammation (Kumar et al., 2015).

#### Autoantibodies

Autoantibodies against brain proteins have been known for some time; recently, they have gained interest in serving as diagnostic tools for CNS injury (Kobeissy and Moshourab, 2015). Disrupted BBB due to TBI permits the leakage of brain proteins and their breakdown products into the circulation, and in some cases, antibodies against these released self-antigens are generated (Raad et al., 2014). Autoantibodies remain in the blood quite a long time, and therefore they present a new class of biomarkers for a past TBI event and chronic sequelae.

Autoantibodies against GFAP and its breakdown products have been recently reported in the context of TBI. When the sera of severe TBI patients were screened using brain immunoblots, a significant increase in the amount of GFAP-specific antibodies was detected beginning at day 5 after TBI (Zhang et al., 2014). The concentrations of GFAP-specific autoantibodies were found to be significantly higher in TBI patients compared with healthy controls at 6 months after injury (Wang et al., 2016). In addition, autoantibodies against S100β were detected in the serum of football players during season (Marchi et al., 2013). The autoantibody levels correlated with the S100β levels measured shortly after each game. The players that were enrolled in the study had suffered regular repeated hits to the head but no concussion or TBI during the game. The authors concluded that even subconcussive hits disrupted the BBB and permitted the leakage of S100β into the blood and subsequent generation of autoantibodies.

### Biomarkers of TBI in clinical laboratories

Of the biomarkers presented in this review, some are available (Table 1) in hospital laboratories, according to the laboratory manuals of large hospitals (Fimlab Laboratories Oy; Hospital District of Helsinki and Uusimaa; Hospital District of Southwest Finland; University of Eastern Finland, Brain Research Unit). Several laboratory assays respond to TBI and other abnormal conditions of CNS. However, S100β is the only one that has TBI as the main indication. The main indications of NSE are neuroblastoma and small cell lung cancer. Tau and Aβ are a biomarkers of Alzheimer’s disease, and cytokines are general biomarkers of inflammation and sepsis. The Scandinavian Neurotrauma Committee has recommended the analysis of serum S100β of head trauma patients who have a mild injury (GCS 14–15) and can be sampled within 6 h after injury (Undén et al., 2013). The concentration of 0.1 µg/l is considered the cutoff for a CT scan (see Discussion). The validation of these guidelines showed that approximately one third of CT scans for mild TBI cases can be avoided with little or no impact on patient outcome (Undén et al., 2015). Diagnostic kits for S100β are available from several manufacturers; however, clinical comparison of kits’ performance has shown that the results are not interchangeable between different suppliers’ assays (Müller et al., 2006; Hallén et al., 2008; Erickson and Grenache, 2011).

**Table 1.** Laboratory tests for the biomarkers reviewed in this article that are available in hospital laboratories.

Biomarker	Sample	Method	Normal range	Range in TBI
S100β	Serum	IC	<0.11 µg/l	>0.11 µg/l^a^
NSE	Serum	Immunodetection based on ECL	From <17 to <25 µg/l, depending on age	>20 µg/l^a^
	CSF	Immunodetection based on ECL	<15 µg/l	54.80 ± 43.34 µg/l^b^
P-tau	CSF	ELISA	<70 pg/ml	N/A
Tau	CSF	ELISA	<400 pg/ml	1684–8691 pg/ml^c^
Aβ-42	CSF	ELISA	>500 pg/ml	<230 pg/ml^d^ <350 pg/ml^e^
IL-6	Plasma	IC	<5.9 ng/l	N/A
IL-8	Plasma	IC	<62 ng/l	N/A
TNF-α	Serum	IC	<8.1 ng/l	N/A

The assays shown in the table respond to the head injuries and to the conditions of the central nervous system, but only S100β has TBI as the main indication. The data were collected from the laboratory manuals of large hospitals in September 2016. IC, immunochemiluminescence; ECL, electrochemiluminescence; Aβ-42, amyloid-beta-42 protein. ^a^Reference values defined in clinical laboratories. ^b^Brandner et al. (2013). ^c^Magnoni et al.(2012). ^d^Franz et al. (2003). ^e^Mondello et al.(2014).

### Nonclassic brain injury markers

The glymphatic system has been suggested to serve as a clearance pathway of interstitial fluid and solutes from the brain parenchyma, and also as a potential route of brain injury biomarkers from the brain to the blood (Iliff et al., 2012; Plog et al., 2015). Interestingly, the pathway itself is impaired after TBI as well. Iliff et al. (2014) found progressive impairment of CSF–interstitial fluid exchange within the glymphatic pathway 1–28 days after TBI. The dysfunction of the glymphatic system results in the accumulation of tau and Aβ, thus promoting the development of neurofibrillary pathology and neurodegeneration. It may be possible to assay the integrity of the glymphatic pathway *in vivo* by using appropriate contrast agents, and this might in the future serve as a highly sensitive novel indicator of brain injury.

## Discussion

We reviewed recent research on TBI biomarkers with special focus on the time course of the markers in easily accessible body fluids relevant for rapid diagnostics. The usual approach in several studies is that the follow-up of the biomarkers starts upon the admission of the patient to the hospital and continues at various intervals for different periods of time, typically a few days to ∼1 week. The admission of the patient to the hospital and the time of the first sampling occurs some time after the accident; thus the first measures in the sequence represent a time point of a few hours after injury at the least. There are hardly any data on the very early kinetics of biomarkers in human subjects because of the lack of rapid tests useful for paramedics and ambulances. Several studies were made on patients who had sustained moderate to severe TBI. Concussions and mTBIs bear less cellular injuries, and the overall release of intracellular molecules is lower, making their measurement more demanding, especially in the blood, because of barriers and dilution, which happens when a molecule traverses from brain to the blood.

**Figure 2.**
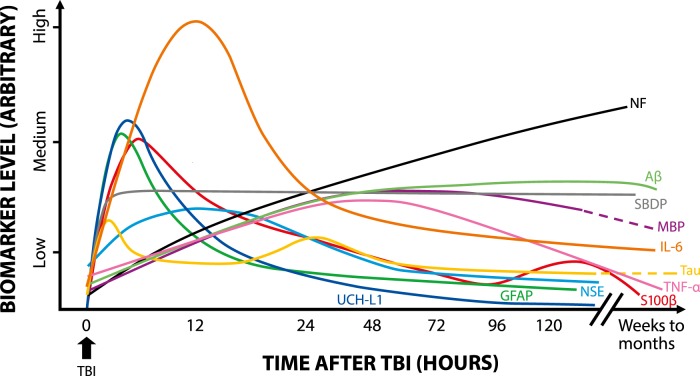
Kinetics of TBI biomarkers. Schematic representation shows the rise and decline of the TBI biomarkers for which representative kinetic data were available in serum or plasma. Separate long-term values (months to weeks) are included when possible.

The time profiles of the biomarkers evidently represent different molecular origins and release mechanisms. Many biomarkers are released during the first burst upon cellular injury and the concomitantly triggered degradation processes. Those markers peak early, within a few hours, and then decline after the molecule-specific half-life in the blood. Neuroinflammation and the emergence of cytokines are somewhat slower processes, and therefore cytokines peak in <48 h. Autoantibodies against brain proteins rise slowly but stay elevated for a fairly long time. The temporal profiles and the relative levels presented in Figure 2 are approximate and must be read with consideration in the absence of uniform data collection and research methods. For example, the severity of TBI affects the peak heights and durations.

Awareness of the temporal profiles of the biomarkers is essential when defining and setting the most appropriate diagnostic time window for sampling after injury. Furthermore, integrated area under the time-curve as a diagnostic determinant, instead of just a single time point measurement, can give advanced diagnostic performance, as shown by Brophy et al. (2011). In addition, the trend between successive measurements indicates the progression of the injury. For example, a TBI patient who was originally considered a mild case showed continuous increase of NSE and S100β until the patient died at 76 h after admission. The mean values of those biomarkers, as calculated from all patients of the group in the study, showed descending trends, however (Herrmann et al., 2000). This is something that frequently remains undisclosed in several study reports; temporal profiles are shown as mean values of the patient cohort or mean values of patient categories (e.g., mild and severe trauma), although follow-up of individual trends would reveal some essential information that is hidden within the mean values.

Recently published Scandinavian guidelines (Undén et al., 2013, 2015) recommend for the first time to measure the biomarker S100β in the serum of patients who have sustained a mild head injury. The biomarker S100β should be assayed in cases where the GCS is 14 and no other risks are present, and when the GCS is 15 and the patient has a history of loss of consciousness and repeated vomiting. The guidelines recommend that the patients mentioned above are admitted to CT imaging only when the concentration of S100β is ≥0.10 µg/l. This approach reduces the number of CT scans by approximately one third and saves those patients from unnecessary exposure to radiation (Undén et al., 2015). The S100β assay has a good negative predictive value (Undén and Romner, 2010; Asadollahi et al., 2015), meaning that a negative value of S100β quite reliably rules out brain injury in any patient. Increased levels of S100β may originate from a brain injury, but also from lesions in some other tissues. This means that a positive value of S100β does not necessarily confirm the presence of a brain injury, especially in multitrauma patients (Sorci et al., 1999; Undén et al., 2013; Gebhardt et al., 2016; Wolf et al., 2016).

TBI, its consequences, and other brain traumas are admittedly gaining increasing awareness in society. The detection of these conditions, as well as the overall assaying of brain status and recovery after injury, is not unambiguous, however. Biomarkers that can be measured from body fluids in regular laboratory practice, or even in decentralized conditions, can supplement diagnosis or perhaps serve as a new means of definitive diagnosis for mild injuries. But, consensus and coherence among TBI biomarkers is still missing, and S100β is the only one that is gradually being implemented into clinical use. Some trends for the future can be seen, however, as diagnostic technologies develop and can detect smaller molecular quantities with higher resolving power. This can bring some current biomarkers into new light. Second, multiplexing—detection of several biomarkers in parallel in one assay—has been adapted in TBI biomarker study as well (Diaz-Arrastia et al., 2014; Di Battista et al., 2015). Furthermore, proteomic (and other “-omic”) approaches can discover new brain injury–related biomolecules which can be harnessed and validated in time into new diagnostic TBI biomarkers.

## Synthesis

Reviewing Editor: Maiken Nedergaard, University of Rochester Medical Center Decisions are customarily a result of the Reviewing Editor and the peer reviewers coming together and discussing their recommendations until a consensus is reached. When revisions are invited, a fact-based synthesis statement explaining their decision and outlining what is needed to prepare a revision will be listed below. The following reviewer(s) agreed to reveal their identity: Benjamin Plog, Peter Bergold.

Your review on biomarkers in TBI was well receved, but both reviewers requested a more detailed describing of inaging biomarkers (glymphatic, fMRI).
